# Sphingomyelin synthase–related protein SMSr is a phosphatidylethanolamine phospholipase C that promotes nonalcoholic fatty liver disease

**DOI:** 10.1016/j.jbc.2023.105162

**Published:** 2023-08-14

**Authors:** Yeun-po Chiang, Zhiqiang Li, Mulin He, Quiana Jones, Meixia Pan, Xianlin Han, Xian-Cheng Jiang

**Affiliations:** 1 Department of Cell Biology, SUNY Downstate Health Sciences University, Brooklyn, New York, USA; 2 Lipidomics Core, The University of Texas Health Science Center at San Antonio, San Antonio, Texas, USA; 3 Molecular and Cellular Cardiology Program, VA New York Harbor Healthcare System, Brooklyn, New York, USA

**Keywords:** sphingomyelin synthase–related protein, phosphatidylethanolamine phospholipase C, phosphatidylethanolamine, nonalcoholic fatty liver disease (NAFLD), nonalcoholic steatohepatitis (NASH), liver fibrosis

## Abstract

Sphingomyelin synthase (SMS)–related protein (SMSr) is a phosphatidylethanolamine phospholipase C (PE-PLC) that is conserved and ubiquitous in mammals. However, its biological function is still not clear. We previously observed that SMS1 deficiency–mediated glucosylceramide accumulation caused nonalcoholic fatty liver diseases (NAFLD), including nonalcoholic steatohepatitis (NASH) and liver fibrosis. Here, first, we evaluated high-fat diet/fructose-induced NAFLD in *Smsr* KO and WT mice. Second, we evaluated whether SMSr deficiency can reverse SMS1 deficiency–mediated NAFLD, using *Sms1/Sms2* double and *Sms1/Sms2/Smsr* triple KO mice. We found that SMSr/PE-PLC deficiency attenuated high-fat diet/fructose–induced fatty liver and NASH, and attenuated glucosylceramide accumulation–induced NASH, fibrosis, and tumor formation. Further, we found that SMSr/PE-PLC deficiency reduced the expression of many inflammatory cytokines and fibrosis-related factors, and PE supplementation *in vitro* or *in vivo* mimicked the condition of SMSr/PE-PLC deficiency. Furthermore, we demonstrated that SMSr/PE-PLC deficiency or PE supplementation effectively prevented membrane-bound β-catenin transfer to the nucleus, thereby preventing tumor-related gene expression. Finally, we observed that patients with NASH had higher SMSr protein levels in the liver, lower plasma PE levels, and lower plasma PE/phosphatidylcholine ratios, and that human plasma PE levels are negatively associated with tumor necrosis factor-α and transforming growth factor β1 levels. In conclusion, SMSr/PE-PLC deficiency causes PE accumulation, which can attenuate fatty liver, NASH, and fibrosis. These results suggest that SMSr/PE-PLC inhibition therapy may mitigate NAFLD.

The sphingomyelin synthase (SMS) gene family includes SMS1, SMS2, and SMS-related protein (SMSr) (1, 2). Unlike SMS1 and SMS2, SMSr lacks SM synthase activity (1, 2), but displays ceramide phosphorylethanolamine (CPE) synthase activity *in vitro* (3). However, gene KO (4, 5) and overexpression (6) studies were unable to confirm this activity *in vivo*. Thus, although SMSr is conserved throughout the animal kingdom and is expressed ubiquitously in mammalian tissues (1, 2), its biochemical activity *in vivo* was unknown until recently.

Mammalian phospholipase C (PLC) is a group of enzymes that cleave phospholipids to produce a diacylglycerol (DAG) and a phosphorylated molecule, such as phosphorylcholine and phosphorylethanolamine (7). We and others found that SMSr has PLC activity (6, 8) and specifically hydrolyzes PE, making it a phosphatidylethanolamine PLC (PE-PLC) (6). We also found that SMS1 and SMS2 are two phosphatidylcholine (PC)-PLCs (9). Given the fact that (10, 11, 12, 13) PC-PLC and phosphatidylinositol (PI)-PLCs (14) maintain PC and PI levels and regulate many important biological functions, SMSr-mediated PE-PLC activity should have an important biological function.

PE is the second most abundant phospholipid in mammalian tissues (15, 16). PE plays an important part in autophagy, cell division, protein folding, and endoplasmic reticulum stress (17, 18, 19, 20). Abnormally high or low PE/PC ratios affect energy metabolism in various tissues and are linked to metabolic diseases, including nonalcoholic fatty liver diseases (NAFLD), insulin resistance, and atherosclerosis (21, 22, 23, 24, 25, 26). Despite its importance, the regulation of PE levels and the mechanisms linking PE changes to metabolic diseases are poorly understood.

A hallmark of NAFLD is triglyceride accumulation in the cytoplasm of hepatocytes, which arises from an imbalance between lipid acquisition and removal (27). The ratio (or index) of triglyceride and glucose in the circulation is a useful marker for screening simple fatty liver and nonalcoholic steatohepatitis (NASH) in humans (28, 29). However, other lipids, including sphingolipids, also have important roles in the development of fatty liver and NASH (30, 31). Ceramides and glycosphingolipids could be two groups of these factors (32, 33). We recently reported that SMS1 deficiency leads to glucosylceramide (GluCer) accumulation, and this is one of the triggers promoting the development of fatty liver into NASH, and subsequently fibrosis and tumorigenesis (34). Since SMSr is a member of the SMS family, we would like to evaluate the effect of SMSr/PE-PLC on GluCer-induced NAFLD.

In this study, we showed that SMSr/PE-PLC deficiency attenuated dietary-induced or GluCer accumulation–induced NAFLD.

## Results

### Effect of SMSr deficiency on high-fat diet/fructose–induced fatty liver and NASH

**Figure 1:**
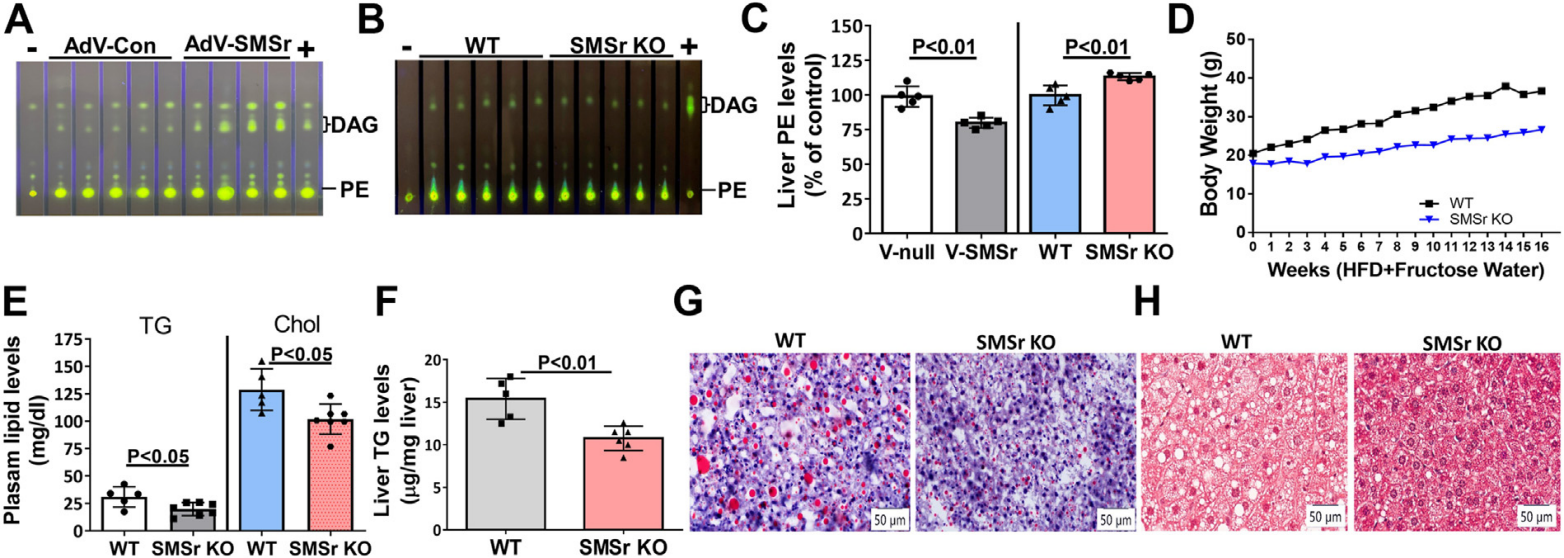
**SMSr deficiency attenuated high-fat diet/fructose–induced fatty liver in 3-month-old WT and *Smsr*-KO mice.** Three-month-old WT and *Smsr*-KO female mice were fed with high-fat diet and fructose in drinking water for 16 weeks. *A*, measurement of liver PE-PLC activity in WT mice treated with adenovirus-SMSr and adenovirus-control. *B*, measurement of liver PE-PLC activity in WT and *Smsr*-KO mice. *C*, PE levels of the liver in WT mice treated with adenovirus-SMSr/adenovirus-control and WT/*Smsr* KO mice. WT and *Smsr*-KO female mice were fed with high-fat diet with fructose (in drinking water) for 16 weeks. *D*, body weight gain measurement, n = 7 per group. *E*, measurement of plasma triglyceride and cholesterol. *F*, measurement of liver triglyceride. *G*, Oil Red O staining in sectioned liver samples; the scale bar represents 50 μm. *H*, trichrome staining in sectioned liver samples; the scale bar represents 50 μm. Data are presented as mean ± SD, n = 5 to 7 per group. The *p* values of (*C*, *E*, and *F*) were calculated by Mann–Whitney *U* test. PE-PLC, phosphatidylethanolamine phospholipase C; SMSr, sphingomyelin synthase–related protein; V-null, adenovirus-control; V-SMSr, adenovirus-SMSr.

We confirmed SMSr is a PE-PLC: administration of adenovirus-SMSr significantly increased PE-PLC activity and decreased PE levels in the liver of WT mice, whereas *Smsr*-KO mice had significantly lower PE-PLC activity and higher PE levels in livers compared with controls (Figs. 1, *A*–*C* and S1, *A* and *B*). To induce fatty liver and NASH, we fed *Smsr*-KO and WT mice with a high-fat diet plus fructose in drinking water for 16 weeks. Starting from week one, *Smsr*-KO mice gained significantly less body weight than WT mice (Fig. 1*D*). SMSr deficiency significantly reduced the levels of plasma triglyceride and cholesterol (Fig. 1*E*). The *Smsr*-KO mice had significantly lower levels of liver triglyceride than WT mice (Fig. 1*F*), which was confirmed by Oil Red O staining of the liver sections (Fig. 1*G*). We also stained the sections with trichrome blue staining. Although we did not observe fibrosis in both sections, we found that SMSr deficiency also reduced lipid droplet size in the liver (Fig. 1*H*). Two enzyme markers for liver damage, alanine transaminase (ALT) and aspartate transaminase (AST), had significantly lower plasma activity levels in *Smsr*-KO mice than in WT mice (Fig. S2).

**Figure 2:**
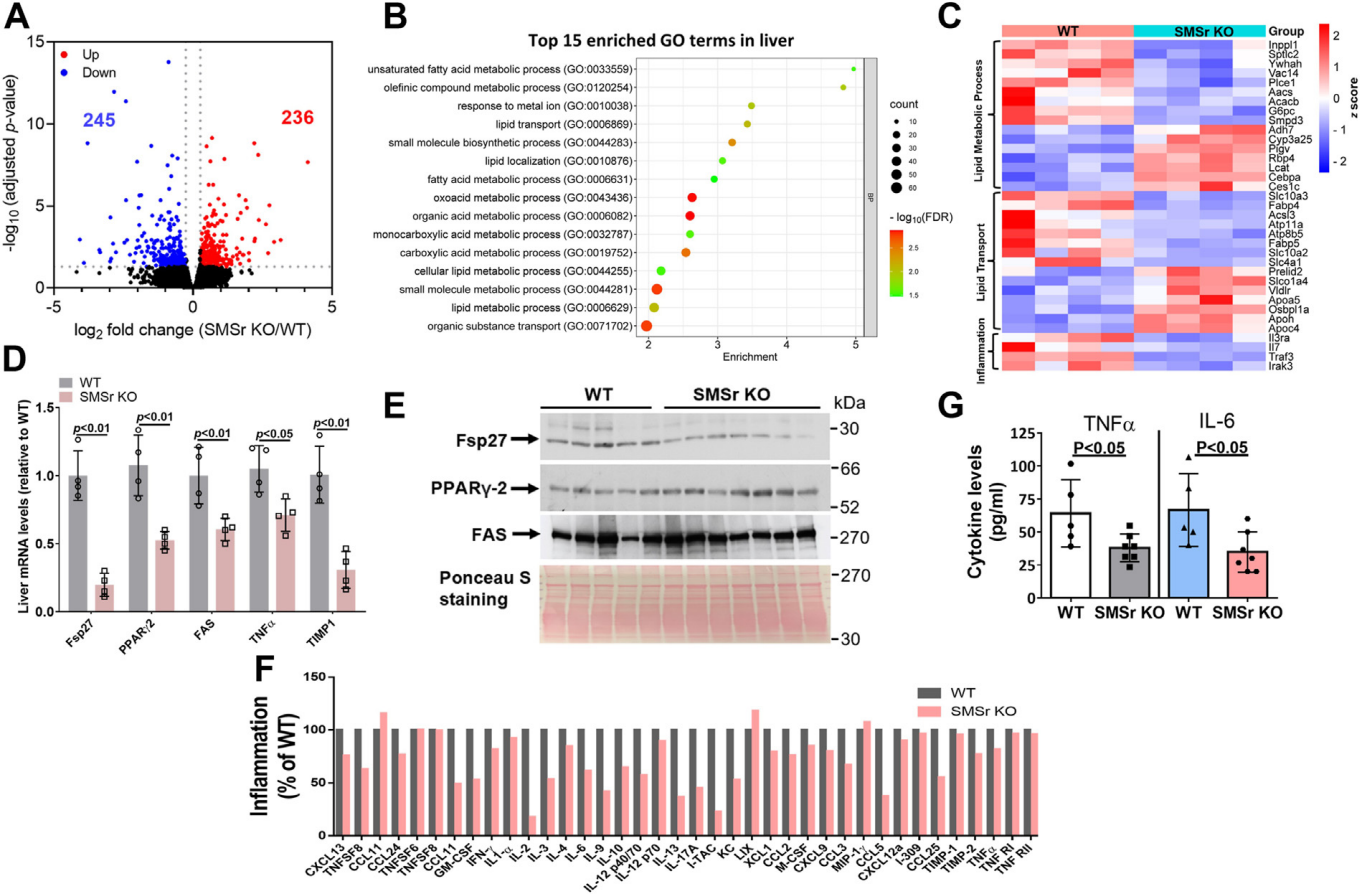
**SMSr deficiency affects hepatic lipid metabolism and inflammation.** Three-month-old WT and *Smsr*-KO female mice were used. *A*–*C*, bulk RNA-seq analysis of liver from WT and *Smsr*-KO mice fed with high-fat diet with fructose (in drinking water) for 16 weeks (n = 4 per group). *A*, volcano plot for differential expression analysis between WT and *Smsr*-KO mice. *B*, Gene Ontology (GO) biological process enrichment analysis of differentially expressed genes in *Smsr*-KO mice liver over WT mice liver. *C*, heatmap of differentially expressed genes related to lipid metabolic process, lipid transport, and inflammation between WT and *Smsr*-KO mice. *D*, real-time PCR analysis of the liver key regulators involved in lipid metabolism. *E*, Western blots for Fsp27 (WT *versus Smsr*-KO, *p* < 0.01), PPARγ-2, and fatty acid synthase (FAS). Ponceau S staining for loading control. *F*, inflammatory cytokine array analysis (*dot blot*). *G*, plasma TNFα and IL-6 levels measured by ELISA. Data are presented as mean ± SD, n = 3 to 7 per group. The *p* values of (*D*, *F*, and *H*) were calculated by Mann-Whitney *U* test. IL-6, interleukin 6; SMSr, sphingomyelin synthase–related protein; TNF, tumor necrosis factor.

Next, we isolated liver from *Smsr* KO and WT mice and performed total RNA-seq. The volcano plot shows that the absence of SMSr in the liver altered the expression of 481 genes (*padj* < 0.05 and∣FC∣>1.5) including 236 upregulated and 245 downregulated genes (Fig 2*A*). Notably, PANTHER GO-slim analysis revealed that lipid metabolism-related biological processes were altered in *Smsr* KO mice (Fig. 2*B*), including fatty acid metabolism, lipid transport, and lipid metabolism. An overview of the top 35 upregulated and downregulated genes is given in Figure 2*C*. These changes could have direct or indirect effects on fatty liver and NASH.

We performed real-time quantitative PCR in liver samples to examine gene expression levels and investigate potential biological mechanisms. The results showed that *Fsp27*, *PPARγ2*, fatty acid synthase (*FAS*), *Tnfα*, and *Timp1* expression levels were significantly reduced in *Smsr*-KO mice than those in WT mice (Fig. 2*D*). No other statistically significant changes in gene expression levels under SMSr/PE-PLC deficiency were detected (Fig. S3). Next, we examined protein levels of Fsp27, FAS, and PPARγ2 in the mouse liver. The results indicated that levels of Fsp27 protein but not FAS or PPARγ2 were significantly reduced (Fig. 2*E*).

We investigated the consequences of fatty liver by performing cytokine array analyses in mouse plasma and found that SMSr/PE-PLC deficiency-induced reductions of about 30 inflammatory cytokines (Fig. 2*F*). We performed ELISA to specifically measure the levels of plasma tumor necrosis factor alpha (TNFα) and interleukin-6 (IL-6), two well-known inflammatory cytokines. The results showed that both were significantly reduced (Fig. 2*H*). These combined results indicate that SMSr/PE-PLC deficiency attenuates high diet/fructose–induced fatty liver and NASH which could be associated with fibrosis or without fibrosis (35). However, we did not observe fibrosis in both WT and SMSr/PE-PLC KO mice.

### Effect of SMSr deficiency on glucosylceramide-induced nonalcoholic steatohepatitis, liver fibrosis, and liver tumor

Our previous study reported that 3-month-old mice with *Sms1* and *Sms2* double KO (dKO) fed with a high-fat diet for 6 weeks had significantly higher lipid accumulation in liver than that of *Sms2*-KO or WT mice (34). Here, we prepared *Sms1*/*Sms2*/*Smsr* triple KO (tKO) mice and then fed WT, *Sms1*/*Sms2*-dKO, and *Sms1*/*Sms2*/*Smsr*-tKO mice (3-month-old, male and female) with high-fat diet for 6 weeks. The results showed that SMSr deficiency did not prevent lipid accumulation (Fig. S4, *A* and *B*).

**Figure 3:**
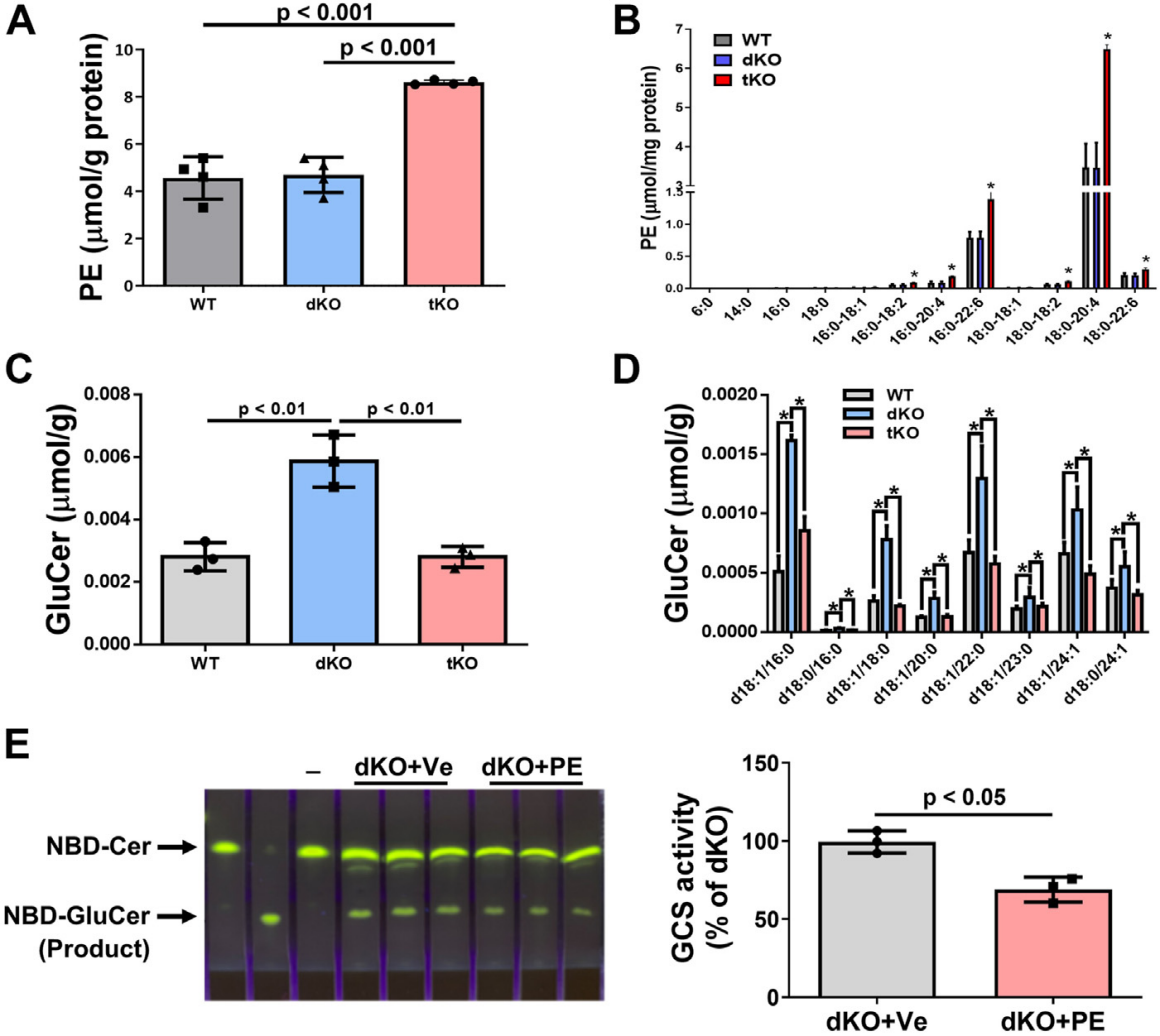
**SMSr deficiency increased liver PE and decreased liver GluCer levels under SMS1/SMS2 double deficiency.** Three-month-old WT and *Smsr*-KO female mice were used. *A* and *B*, total and individual PE levels in the liver among WT, *Sms1/Sms2*-dKO, and *Sms1*/*Sms2/Smsr*-tKO mice. *C* and *D*, total and individual GluCer levels in the liver among WT, *Sms1/Sms2*-dKO, and *Sms1*/*Sms2/Smsr*-tKO mice. *E*, GluCer synthase measurement. *Sms1/Sms2*-double KO mice were treated with PE or vehicle for 7 days and liver GluCer synthase activity was measured and quantified. Data are presented as mean ± SD, n = 3 to 4 per group. The *p* values of (*A*–*D*) were calculated by Kruskal–Wallis test followed by Mann–Whitney pairwise tests (*A* and *C*) or Dunn post hoc multiple comparisons tests (*B* and *D*). The *p* values of (*E*) were calculated by Mann–Whitney *U* test. ∗*p* < 0.05, ∗∗*p* < 0.01. GluCer, glucosylceramide; PE, phosphatidylethanolamine; SMSr, sphingomyelin synthase–related protein; tKO, triple KO.

Next, we utilized the dKO and the tKO mice on chow diet to perform the rest of the experiments. We measured different lipid levels in the liver of the dKO and the tKO mice and confirmed that there were no differences in sphingomyelin and ceramide (9) (Fig. S5). We also measured different subspecies of PE in the liver of WT, dKO, and tKO mice, and found that total and almost all tested individual PEs were significantly higher in tKO mice than in WT or dKO mice (Fig. 3, *A* and *B*). We confirmed that the dKO mice had significantly higher GluCer levels in the liver than the WT mice (34) (Fig. 3, *C* and *D*). Importantly, the tKO mice had significantly lower GluCer levels in the liver than the dKO mice (Fig. 3, *C* and *D*), which could be due to SMSr deficiency–mediated PE accumulation. We then treated the dKO mice with PE and found that PE supplementation significantly reduced glucosylceramide synthase (GCS) activity (Fig. 3*E*). These combined results suggest that SMSr/PE-PLC deficiency–mediated PE induction could reduce GCS activity thereby reducing GluCer levels in the liver.

**Figure 4:**
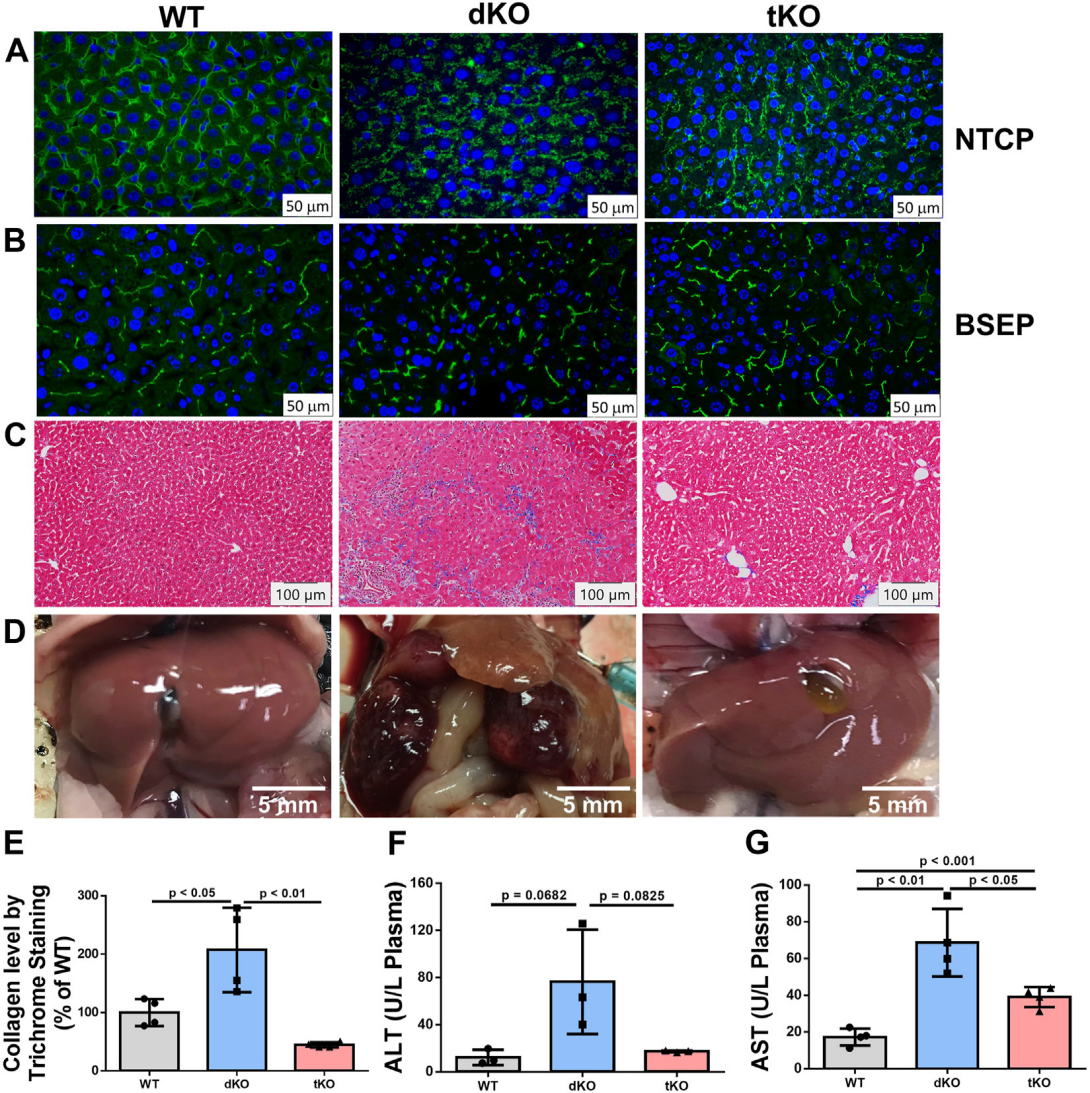
**SMSr deficiency reversed SMS1 deficiency–mediated liver impairment.** Liver sections from 6-month-old WT, *Sms1/Sms2*-dKO, and *Sms1*/*Sms2/Smsr*-tKO female mice were used for this analysis. *A*, immunostaining of hepatocyte basal membrane for Na^+^-taurocholate cotransporting polypeptide (NTCP). *B*, immunostaining of hepatocyte apical membrane for bile salt export pump (BSEP). *C* and *E*, trichrome staining for fibrosis and quantification. *D*, liver tumor observation in 1-year-old WT, *Sms1*/*Sms2*-dKO, and *Sms1*/*Sms2*/*Smsr*-tKO mice. All the dKO mice(6/6) developed tumors with various sizes and none WT and the tKO mice had tumor. Pictures are representative of six mice/group. *F* and *G*, fluorogram of plasma alanine aminotransferase (ALT) and aspartate aminotransferase (AST) levels. Data are presented as mean ± SD, n = 3 to 4 per group. The *p* values of (*E*–*G*) were calculated by Kruskal-Wallis test followed by Mann-Whitney pairwise tests. dKO, double KO; SMSr, sphingomyelin synthase–related protein; tKO, triple KO.

Our previous study observed liver abnormalities in a 6-month-old dKO mice that were on a chow diet compared with WT and *Sms2*-KO mice (34). The current study utilized mice that were ≥6-months old. We visualized hepatocyte basal membranes in WT, the dKO, and the tKO mice by immunostaining with Na^+^-taurocholate co-transporting polypeptide (NTCP), which is a hepatocyte basal membrane marker (36). NTCP was precisely localized in WT hepatocyte basal membranes, whereas NTCP displayed diffuse cytosolic localization in the dKO hepatocytes and this abnormality was corrected by the tKO hepatocytes, at least partially (Fig. 4*A*). We also stained for bile salt export pump, which is located on the bile canaliculi membrane (apical membrane) (37). In normal liver section, bile canalicular should be stained as dots (cross section) and networks (longitudinal section). We can see the number of networks is more than that of dots in WT and the tKO mouse liver sections (Fig. 4*B*). However, the networks are greatly reduced in the dKO mouse liver sections (Fig. 4*B*), suggesting an impairment. Also, we measured total bile acids and found that the WT and tKO mice had significantly lower bile acids than that of the dKO mice (Fig. S6). Collectively, SMSr deficiency reversed the liver impairment, at least partially.

We stained 6-month-old female mouse liver sections with trichrome and found that the dKO but not WT or the tKO mouse livers had fibrosis (Figs. 4*C* and S7). We quantified collagen fibers stained with trichrome and found that the dKO mouse livers had significantly higher levels of collagen fibers than that of WT mice and this effect was greatly reversed in the tKO mouse livers (Fig. 4*E*), indicating that SMSr deficiency could reverse liver fibrosis caused by GluCer accumulation (34). We observed that the dKO mice suffered from liver cirrhosis, and it was technically challenging to obtain primary hepatocytes using the collagenase perfusion procedure when the dKO mice were 4 months or older. By contrast, we had no difficulties obtaining primary hepatocytes from the tKO mouse livers, regardless of age. One obvious pathology of 1-year-old dKO mice was that they had liver tumors of various sizes, whereas 1-year-old WT or the tKO mice did not have any tumors (Figs. 4*D* and S8). Similar phenotypes were observed in male mice (data not shown). We also measured ALT and AST in plasma and found that the tKO mice had significantly lower ALT and AST activity levels than that of the dKO mice (Fig. 4, *F* and *G*).

### Effect of SMSr deficiency on the expression of genes related to inflammation, fibrosis, and tumorigenesis

**Figure 5:**
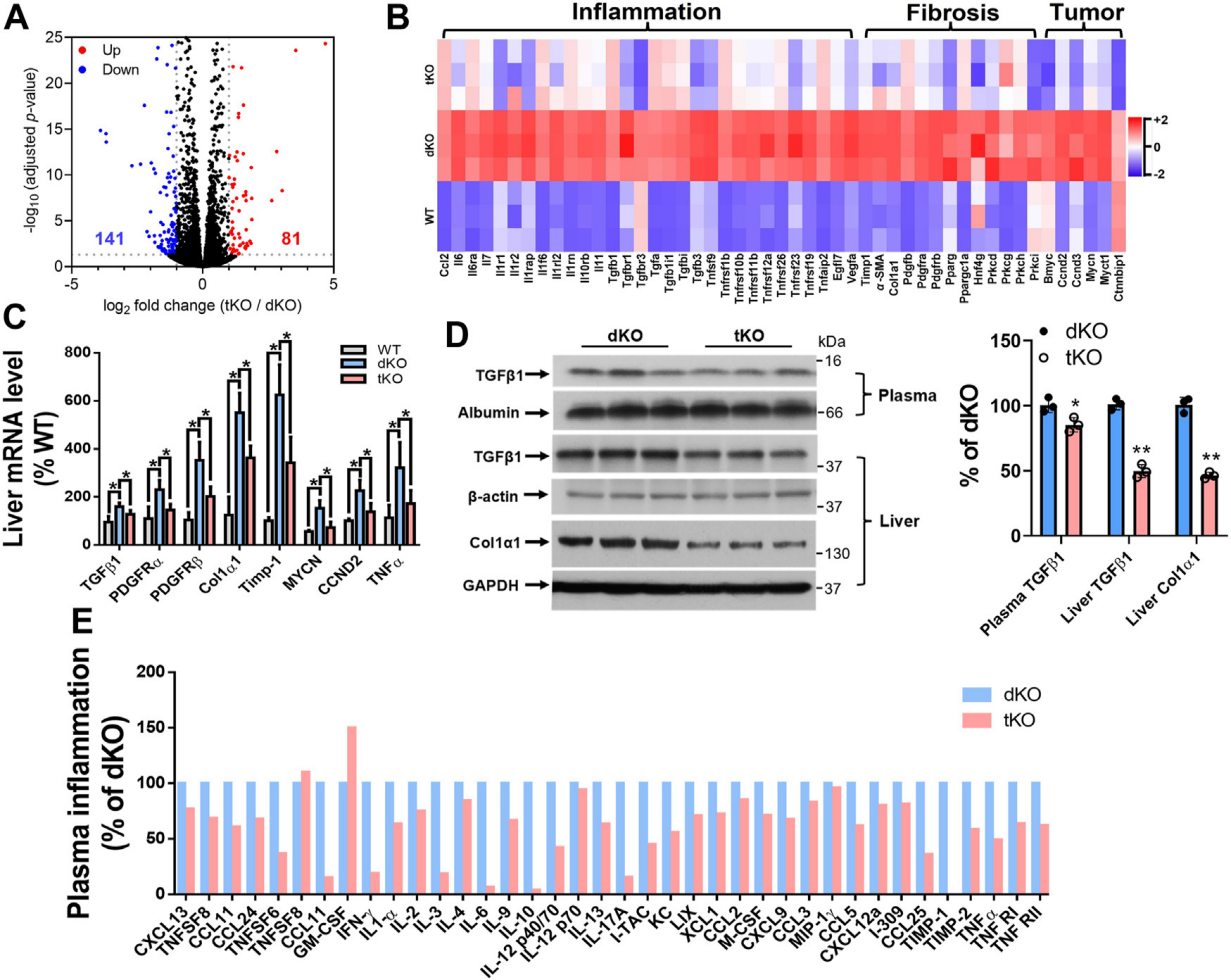
**SMSr deficiency reversed SMS1 deficiency–mediated NASH, liver fibrosis, and liver tumor formation.** We isolated primary hepatocytes from chow-fed 2-month-old WT, *Sms1*/*Sms2*-dKO and *Sms1*/*Sms2*/*Smsr*-tKO female mice. *A* and *B*, bulk RNA-seq analysis of hepatocytes isolated from WT, dKO, and tKO mice (n = 3 per group). *A*, volcano plot for differential expression analysis between *Sms1/Sms2*-dKO and *Sms1/Sms2/Smsr*-tKO mice. *B*, heatmap for inflammation, fibrosis, and tumorigenesis-related genes in WT, *Sms1/Sms2*-dKO, and *Sms1/Sms2/Smsr*-tKO mouse hepatocytes. *C*, real-time PCR analysis of the liver key regulators involved in inflammation and fibrosis among WT, *Sms1/Sms2*-dKO, and *Sms1/Sms2/Smsr*-tKO mice. *D*, Western blot analysis of TGFβ1 (plasma and liver) and collagen 1α1 (liver) between *Sms1/Sms2*-dKO and *Sms1/Sms2/Smsr*-tKO mice. *E*, inflammatory cytokine array analysis (dot blot). Data are presented as mean ± SD, n = 3 to 4 per group. The *p* values of (*C*) were calculated by Kruskal–Wallis test followed by Mann–Whitney pairwise tests. The *p* values of (*D*) were calculated by unpaired two-tailed Student’s *t* test. ∗*p* < 0.05, ∗∗*p* < 0.01. dKO, double KO; NASH, nonalcoholic steatohepatitis; SMSr, sphingomyelin synthase–related protein; TGFβ1, transforming growth factor β1; tKO, triple KO.

We reported previously that the dKO mouse liver expressed many inflammation-, fibrosis-, and tumorigenesis-related genes, such as upregulation of transforming growth factor β1 (*Tgfβ1*), *Tnfα*, *Il6, Il1*, *Colα1*, *Pdfgrs*, and *Mycn* compared with WT mice and we attributed this to SMS1 deficiency–mediated GluCer accumulation (34). In current study, we isolated primary hepatocytes from chow-fed dKO and tKO female mice and performed total RNA-seq. We found that, in comparison with the dKO hepatocytes, the tKO hepatocytes have 81 upregulated genes and 141 downregulated genes (Fig. 5*A*). Among the 141 downregulated genes, we listed 53 which are related with NASH, fibrosis, the tumorigenesis, together with that of WT mice (Fig. 5*B*). We performed real-time PCR for *Tgfβ1*, *Tnfα, Colα1*, *Pdfgrs*, *Timp-1*, *CCND2*, and *Mycn*, the key factors for NASH, liver fibrosis, and liver tumor (38) in WT, the dKO, and the tKO mouse livers. All showed similar trends of increased expression in livers from the dKO mice that were reversed, at least partially, to WT levels in the tKO mice (Fig. 5*C*). We performed immunoblotting for TGFβ1 (a 43 kDa precursor and a 12.5 kDa active form) and collagen 1α1 in the liver. The levels of all proteins were significantly decreased in the tKO mouse livers than those in the dKO mouse livers (Fig. 5*D*). Inflammatory cytokine array analysis indicated that approximately 20 cytokines were downregulated in the tKO plasma than that in the dKO plasma (Fig. 5*E*). These combined results indicate that SMSr deficiency attenuates NASH and fibrosis caused by a deficiency of both SMS1 and SMS2.

### PE attenuates TGFβ1-mediated fibrosis and tumorigenesis

**Figure 6:**
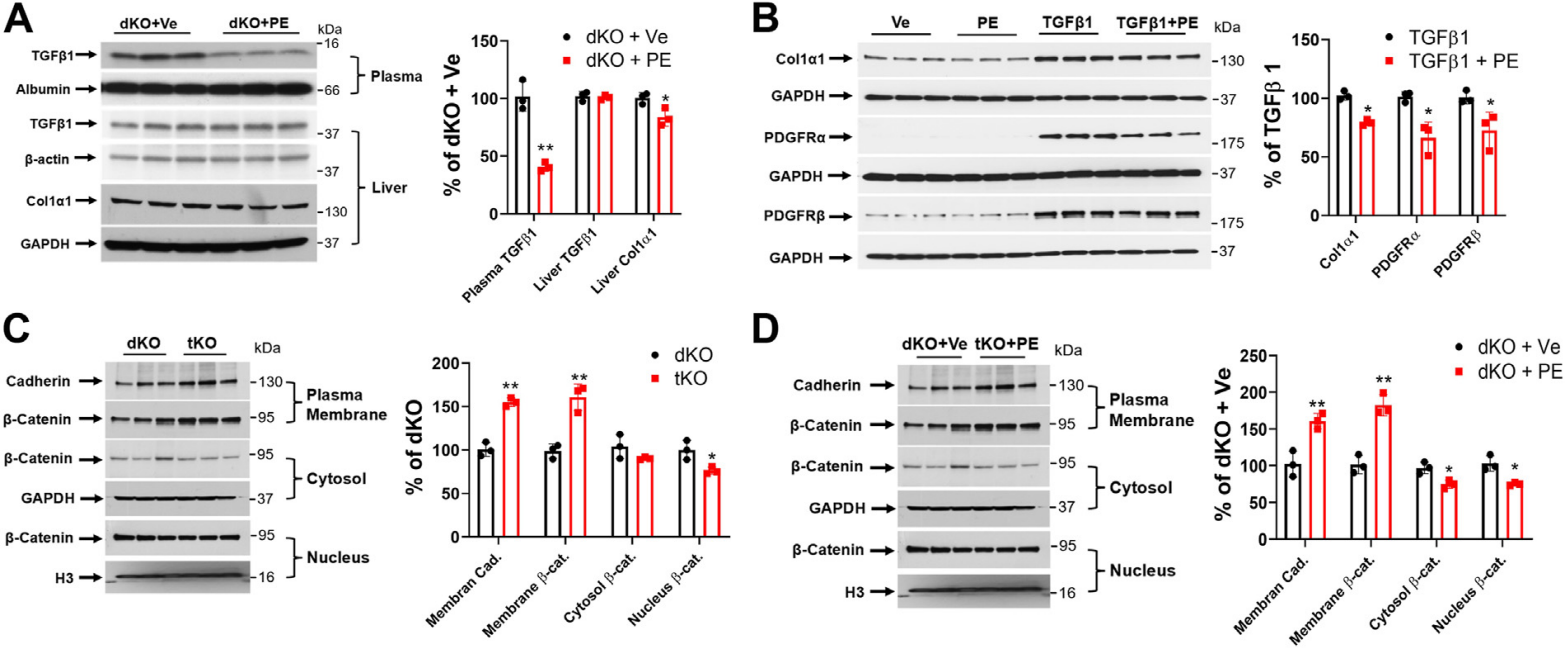
**SMSr-PE-PLC deficiency or PE supplementation reversed the effects of SMS1 deficiency on TGFβ1, Col1α1, Cadherin, β-catenin, and PDGFRs.***A*, three-month-old *Sms1/Sms2*-dKO mice were treated with PE or vehicle for 7 days before measuring plasma TGFβ1, liver TGFβ1, and Col1α1 by Western blot and quantified. *B*, LX2 cells were treated with vehicle, PE (20 μM), TGFβ1 (10 ng/ml), or TGFβ1 plus PE. Cell Col1α1 and PDGFRs were measured by Western blot and quantified. *C*, Western blot analyses of cadherin and β-catenin in liver plasma membrane, cytoplasm, and nucleus of *Sms1/Sms2*-dKO and *Sms1/Sms2/Smsr*-tKO mice. Quantification was shown by fluorogram. *D*, Western blot analyses of cadherin and β-catenin in liver plasma membrane, cytoplasm, and nucleus of *Sms1/Sms2*-dKO mice treated with vehicle or PE. Quantification was shown by fluorogram. Data are presented as mean ± SD, n = 3 per group. The *p* values of (*A*–*D*) were calculated by unpaired two-tailed Student’s *t* test. ∗*p* < 0.05, ∗∗*p* < 0.01. PDGFR, platelet-derived growth factor receptor; PE-PLC, phosphatidylethanolamine phospholipase C; SMSr, sphingomyelin synthase–related protein; TGFβ1, transforming growth factor β1; tKO, triple KO.

To observe the direct effect of PE (mimicking SMSr deficiency), we treated the dKO mice with PE (i.p.) (10 mg/kg) every day for 7 days to mimic triple deficiency. Immunoblotting analysis showed that the active form of TGFβ1 (12.5 kDa) was dramatically reduced in PE-treated dKO mouse plasma (Fig. 6*A*). In PE-treated dKO liver, there was no change in the level of TGFβ1 precursor (43 kDa), but collagen 1α1 was significantly reduced (Fig. 6*A*), suggesting that PE supplement–mediated reduction of TGFβ1 in the plasma could mitigate liver fibrosis.

We next examined the effects of PE supplementation on LX2 cells, a hepatic stellate cell (HSC) line, by treating the cells with TGFβ1 (10 ng/ml) with or without 20 μM PE. The results showed that PE supplementation in LX-2 cells significantly reduced TGFβ1-induced production of fibrosis proteins (*i.e.*, collagen 1α1, platelet-derived growth factor receptor (PDGFR)α, and PDGFRβ) (Fig. 6*B*), suggesting that PE supplementation could rescue liver fibrosis caused by TGFβ1 stimulation.

We found that the dKO but not WT and the tKO mice develop liver tumors (Figs. 4*D* and S6). This could be related to the TGFβ1-Wnt/β-catenin pathway (39, 40). TGFβ1 upregulation in *Sms1*/*Sms2*-dKO liver could result in β-catenin translocation from hepatocyte plasma membrane into the nucleus, thereby initiating tumorigenesis (34). We detected significant accumulation of β-catenin in the plasma membrane of the tKO mouse liver and a significant reduction of β-catenin in the corresponding cytosol and nucleus (Fig. 6*C*) compared with the levels in the *Sms1*/*Sms2*-dKO mouse liver. We also detected significant accumulation of cadherin in the plasma membrane of the tKO liver (Fig. 6*C*). This result indicates that SMSr deficiency could reverse TGFβ1-Wnt/β-catenin-mediated liver tumorigenesis. We injected the dKO mice with PE (i.p.) to confirm these results and found that PE treatment increased β-catenin and cadherin on liver plasma membrane and decreased β-catenin in the cytosol and nucleus compared with the levels in the control (Fig. 6*D*). This result indicated that PE can reduce cytosolic and nuclear levels of β-catenin.

### Association of liver SMSr/PE-PLC activity and plasma PE with human NASH

**Figure 7:**
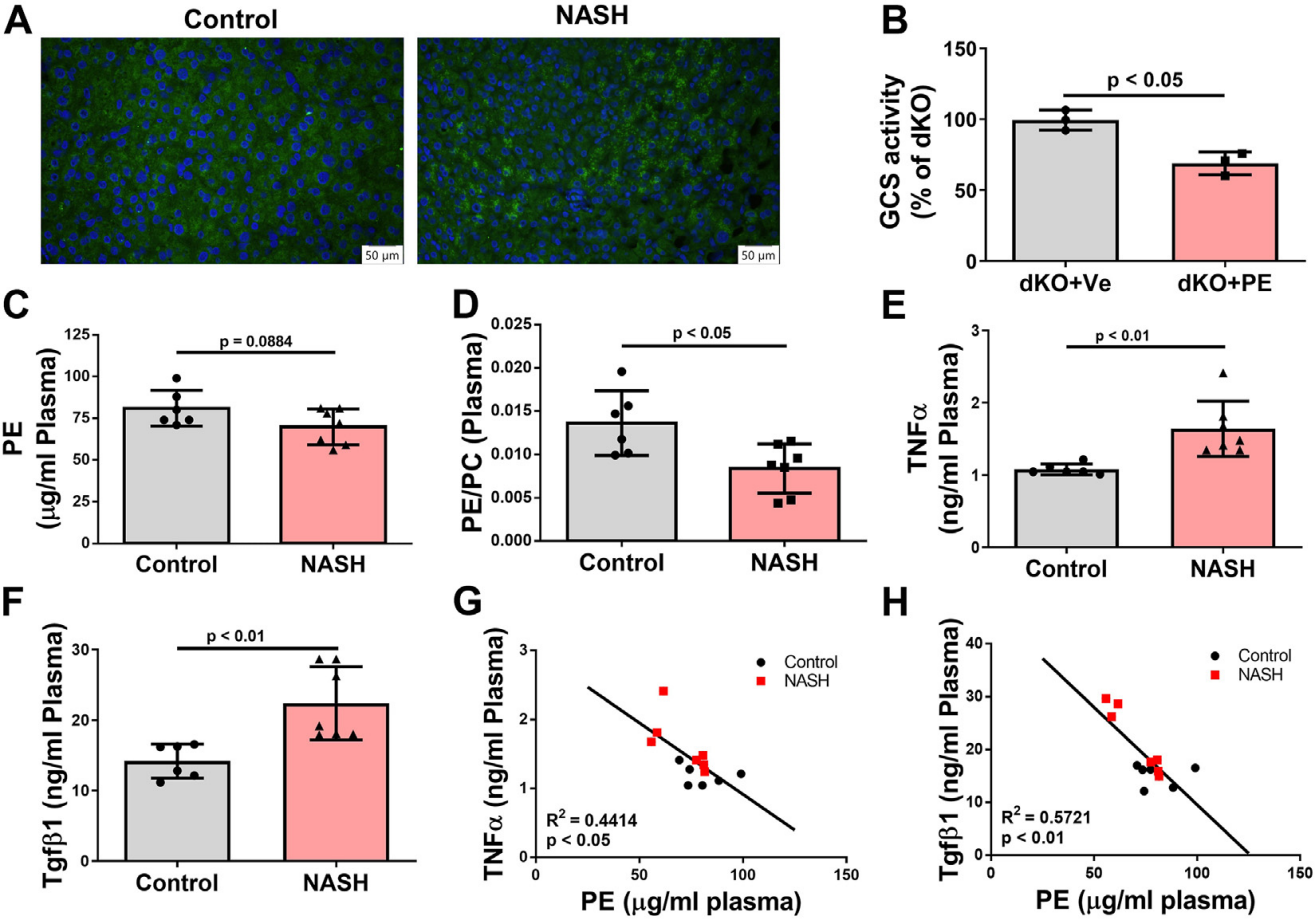
**SMSr/PE-PLC and PE levels in NASH patients.** Human liver microarrays with normal and NASH sections were purchased from Sekisui XenoTech, LLC. Human female NASH plasma and control plasma were purchased from Discovery Life Sciences. *A* and *B*, SMSr immunostaining was performed on liver sections from human NASH patients and controls. Quantification was shown by fluorogram. *C* and *D*, NASH patient and control plasma PE and PC were measured. PE and PE/PC quantification was shown by fluorogram. *E* and *F*, NASH patient and control plasma TNFα and TGFβ were measured by ELISA. *G*, correlation between human plasma PE and TNFα levels. *H*, correlation between human plasma PE and TGFβ1 levels. Data are presented as mean ± SD, n = 4 to 7 per group (*B*–*F*). The *p* values of (*B*–*F*) were calculated by unpaired two-tailed Student’s *t* test. The *p* values of (*G* and *H*) were calculated by linear regression model. NASH, nonalcoholic steatohepatitis; PC, phosphatidylcholine; PE-PLC, phosphatidylethanolamine phospholipase C; SMSr, sphingomyelin synthase–related protein; TGFβ1, transforming growth factor β1; TNF, tumor necrosis factor.

We evaluated the relationship between PE-PLC/SMSr and NASH in humans. Liver samples from NASH patients and controls were immunostained with SMSr antibody. SMSr protein expression was significantly higher in the diseased livers (Fig. 7, *A* and *B*). NASH patients displayed a trend of reduced PE in the plasma, but it was not statistically significant (Fig. 7*C*). We also measured PC levels and found that the difference was not significant (Fig. S9*A*). However, the plasma PE/PC ratio was significantly lower in NASH patients than in controls (Fig. 7*D*). We also found that NASH patients had significantly higher levels of plasma TNFα and TGFβ1 (Fig. 7, *E* and *F*). Importantly, plasma TNFα levels were negatively correlated with PE (Fig. 7*G*), as were TGFβ1 and PE (Fig. 7*H*). The correlation of PC with TNFα or TGFβ was not significant (Fig. S9, *B* and *C*). The combined results indicate that plasma PE levels are negatively associated with human NASH.

## Discussion

This is the first functional study of SMSr as a PE-PLC. First, we provide evidence that in comparison with WT mice, SMSr/PE-PLC deficiency can attenuate high fat/fructose-induced NAFLD, including fatty liver and NASH. Second, the current study is a continuation of our previous study (34) which indicated that the SMS1 deficiency-mediated GluCer accumulation in mice results in NAFLD, including fatty liver, NASH, and liver fibrosis as well as tumor. We found that the SMSr deficiency can reverse GluCer accumulation-mediated NASH, liver fibrosis, and tumor formation and provided evidence that PE-PLC/SMSr deficiency–mediated PE accumulation is the key factor mediating these beneficial effects.

SMSr transfers phosphorylethanolamine from PE to ceramide *in vitro* to produce CPE (3). Although *Smsr* is expressed ubiquitously (4), we and others showed that CPE levels are extremely low in the liver and plasma (4, 5) and undetectable in most mammalian tissues (unpublished observation). Mouse *Smsr* gene KO (4, 5) or overexpression (6) had little effect on CPE. Therefore, mammalian SMSr is not a functional CPE synthase *in vivo*. The current study is an investigation of the effect of SMSr/PE-PLC–mediated PE changes on NAFLD.

PE can be regulated by SMSr/PE-PLC. Tissue PE levels are controlled by biosynthesis and catabolism. In addition to a common biosynthesis pathway (the Kennedy pathway) (16), mammalian tissues have PC-specific PLCs and PI-specific PLCs that hydrolyze PC and PI to regulate steady-state levels of PC and PI (14, 41, 42). We recently reported that SMSr can hydrolyze PE in the absence of ceramide, and SMSr expression in mouse liver can affect PE levels (6). We confirmed that SMSr/PE-PLC overexpression significantly induced liver PE-PLC activity and reduced liver PE levels (Fig. 1, *A* and *C*), whereas SMSr/PE-PLC deficiency significantly reduced liver PE-PLC activity and increased liver PE levels (Fig. 1, *B* and *C*). We observed that SMSr/PE-PLC deficiency did not completely eliminate PE-PLC activity (Fig. 1*B*), suggesting that other enzymes can hydrolyze PE to generate DAG. PE is essential for autophagy, cell division, and protein folding, and is a precursor for several protein modifications (17, 18). PE promotes nonlamellar membrane structures and modulates membrane curvature and fluidity (43). Abnormally high or low PE/PC ratios affect energy metabolism in various tissues and are linked to disease progression (21).

Although PE is important in several aspects of metabolic diseases, the mechanisms linking PE to metabolic diseases are mostly unknown. We observed that SMSr/PE-PLC deficiency–mediated PE accumulation in liver attenuated high-fat diet/fructose–induced body weight gain, hyperlipidemia, fatty liver, and NASH. Liver RNA-seq analysis clearly indicates that SMSr deficiency is closely related with lipid metabolic process, lipid transport, and inflammation (Fig. 2, *B* and *C*). We then evaluated mRNA levels of certain key factors, including PPARγ2, FAS, Fas27, and TNFα in NAFLD and found that SMSr deficiency can significantly reduce all of them. Further study indicated that Fsp27, TNFα, IL-6, and more than 20 inflammatory cytokines were reduced (Fig. 2, *D*–*F*). Fsp27 is expressed in the livers of diet-induced obese mice (44, 45). Fsp27 inhibition mitigates diet-induced NASH in mice (46). TNFα and IL-6 have been identified as a major regulator of inflammatory responses and is involved in the pathogenesis of many inflammatory diseases (47), and they participate in the development and progression of NAFLD (48). However, we did not observe fibrosis in both WT and SMSr/PE-PLC deficient mice. The stages of NASH-associated fibrosis range from absent (stage F0) to cirrhosis (stage 4), with stages F2—F4 considered to be clinically significant and stages F3—F4 considered to be advanced fibrosis (35). The reason that we did not observe fibrosis in both WT and the KO mice could be due to the feeding time not long enough.

We recently reported that blocking the liver SM synthase (SMS1 and SMS2 double deficiency) caused NASH, fibrosis, and liver tumor. All these phenotypes were related to GluCer accumulation caused by SMS1 deficiency (34). We hypothesized that SMSr/PE-PLC deficiency could reverse the metabolic phenotypes observed in SMS1/SMS2 double-deficient mice. Therefore, we prepared SMS1/SMS2/SMSr triple-deficient mice, and observed that triple deficiency increased liver total PE (Fig. 3*A*) and individual PE (Fig. 3*B*) compared with the SMS1/SMS2 double deficiency. We also found that the triple deficiency significantly reduced liver GluCer levels (Fig. 3, *C* and *D*), and PE supplementation *in vivo* significantly inhibited GCS activity in the liver (Fig. 3*E*). Many studies report that GCS inhibition mitigates hepatic steatosis in mouse models (33, 49, 50), and GCS is highly expressed in the liver of NASH patients (34). Simple fatty liver is considered benign, but the initiation of inflammation and subsequent fibrosis and cirrhosis are critical steps in NAFLD with NASH (51, 52, 53). SMSr could be a new therapeutic target for these debilitating diseases.

RNA-seq and protein dot blot analysis indicated that more than 20 inflammatory cytokines, including TNFα and TGFβ1, were upregulated in the dKO mouse livers compared with WT livers, and the tKO brought these cytokine levels into normal ranges (Fig. 5, *B* and *C*). TGFβ1 is an important cytokine that performs many cellular functions, including inflammation, fibrosis, and tumorigenesis (54). The TGFβ1 precursor has 391 amino acids, with a molecular weight of ∼43 kDa (55). The mature form of TGFβ1 is a dimer (∼25 KDa) with a disulfide bond that can be reduced to form a secretory molecule of TGFβ1 with a molecular weight of ∼12.5 kDa (55). The tKO mice had significantly lower levels of the active form of TGFβ1 in the circulation, and significantly lower collagen 1α1, which is directly associated with fibrosis, in the liver than those of dKO mice (Fig. 5*D*). Administration of PE to the dKO mice significantly reduced TGFβ1 and collagen 1α1 levels (Fig. 6*A*). PE-treated HSC significantly attenuated TGFβ1-mediated upregulation of collagen 1α1 and PDGFR α and β (Fig. 6*B*), which are key factors for liver fibrosis (56). Thus, SMSr deficiency or PE administration effectively prevented TGFβ1-mediated overexpression of liver fibrosis genes.

Why does PE accumulation prevent the transition from fatty liver into NASH and fibrosis? PE improves the processing of glycosylphosphatidylinositol--associated proteins (57). glycosylphosphatidylinositol moieties have crucial roles in driving transient, relatively ordered membrane domains rich in sphingolipids and cholesterol (called lipid rafts) to their target regions (58, 59, 60). PE also reduces endoplasmic reticulum (61) stress, which is closely related to NASH and liver fibrosis (62). Autophagy is a critical pathway for the degradation of intracellular components by lysosomes. Autophagy can regulate hepatocellular lipid accumulation by selective degradation; thus, autophagy has been implicated in a protective role during NAFLD and NASH (63). PE increases autophagic flux because autophagy depends on PE for autophagosome formation (64).

Another key finding of the current study was that SMSr deficiency prevented tumorigenesis caused by GluCer accumulation (Figs. 4*D* and S6). It is known that only a small portion of liver fibrosis can result in tumors (52, 65) and TGFβ1 is one of the factors promoting this transformation (39, 40). TGFβ1 can disrupt the cell plasma membrane cadherin/β-catenin complex and promote β-catenin nuclear translocation (66). We previously showed that liver SMS1 deficiency affects the cellular distribution of β-catenin, which is transferred from the plasma membrane into the nucleus, to stimulate tumor gene expression (34). We found that the tKO mice and PE-supplemented dKO (mimicking the tKO) mice displayed significant disruption of β-catenin transfer to the nucleus (Fig. 6, *C* and *D*), which was typically observed in the dKO mice (34). Thus, SMSr deficiency or PE administration effectively prevented the transition of liver fibrosis to the development of liver tumor.

The field of cancer therapy has different views about PE. The plasma membrane of normal cells is an asymmetric lipid bilayer with PE in the inner leaflet, whereas PE is located in the outer leaflet in cancer cells (67). PE-binding peptides and some small molecules (*e.g.*, ophiobolin A) bind to PE and induce pore formation, subsequently leading to cell lysis (68). It has been suggested that targeting PE on endothelial cells in tumor vasculature could achieve antiangiogenesis and inhibit cancer development (67). Although we cannot fully explain our observation that SMSr deficiency–mediated PE accumulation prevented rather than promoted tumorigenesis, PE accumation related to the enhencement of β-catenin menbrane binding could have a dominant effect. This deserves further study.

To evaluate the relevance of the current study for human NASH, we performed immunostaining to examine SMSr protein mass in the livers of NASH patients and controls. We found that NASH patients had significantly higher SMSr protein mass compared with controls (Fig. 7, *A* and *B*). We also found that NASH patients had lower plasma PE and PE/PC ratio, although the former was not statistically significant (Fig. 7, *C* and *D*). The plasma levels of two inflammatory cytokines, TNFα and TGFβ1, were significantly higher in NASH patients compared with controls (Fig. 7, *E* and *F*), and both were negatively associated with plasma PE levels (Fig. 7, *G* and *H*).

However, the relationship between liver PE and NAFLD is controversial. On the one hand, liver PE levels were reduced in NAFLD (69). On the other hand, elevated liver PE levels increased susceptibility to the disease progression of obesity associated NAFLD (70). The controverse also exist on PE/PC ratio. Decreasing or increasing PE/PC ratio can result in NAFLD (71). Even increased plasma levels PE were associated with NAFLD (72). Thus, there must be a proper ratio of both phospholipids in the circulation; otherwise, the metabolic balance could be compromised and NAFLD/NASH could be promoted. Although we cannot provide the proper ratio from current study, plasma PE seems to be a protective factor for the development of NASH.

In summary, we found that SMSr/PE-PLC deficiency caused PE accumulation, which can attenuate NAFLD, including fatty liver, NASH, liver fibrosis, and liver tumor formation, under different conditions. This suggests that inhibition of SMSr/PE-PLC could be a new strategy for the treatment of NAFLD.

## Experimental procedures

### Mice

We have *Smsr*-KO mice (4). We crossed *Sms1*-Flox mice with global *Sms2*-KO mice, yielding homozygous *Sms1*-Flox/*Sms2*-KO mice. Then, we crossed *Sms1*-Flox/*Sms2*-KO mice with global *Smsr*-KO mice to yield *Sms1*-Flox/*Sms2*/*Smsr*-KO mice. Finally, we crossed *Sms1*-Flox/*Sms2*-KO mice and *Sms1*-Flox/*Sms2*/*Smsr*-KO mice with albumin-Cre transgenic mice, respectively, to obtain liver-specific *Sms1*/global *Sms2*-dKO mice (73) and liver-specific *Sms1*-KO/global *Sms2/*global *Smsr*-tKO mice (6, 9). We used male and female mice that had a C57BL/6 genetic background. Experiments involving animals were conducted with the approval of the Institutional Animal Care and Use Committee at SUNY Downstate Medical Center.

### Adenovirus administration

Adenovirus-SMSr (AdV-SMSr) was prepared commercially (ViraQuest Inc). Mice were injected (i.v.) with AdV-SMSr (1 × 10^11^ viral particles/mouse), respectively. AdV-null was used as the control. On day four after the injection, mouse livers were collected for enzyme and lipid analyses.

### High-fat diet/fructose study

Three-month-old mice were fed with a high-fat diet (Envigo, Cat# TD88137) with or without fructose in drinking water for 16 weeks (5–6 mice each group). Mouse body weight was measured every week. Plasma triglyceride (Triglyceride kit, Thermo Fisher Scientific), plasma cholesterol (Cholesterol kit, Fujifilm), and liver triglyceride were measured.

### Real-time PCR

Total RNA was isolated from the liver using TRIzol according to the manufacturer’s instructions. Complementary DNA was synthesized using high-capacity complementary DNA reverse transcription kits (Applied Biosystems). Real-time PCR was performed on the StepOnePlus Real-time PCR system using the SYBR Green Master Mix System (Applied Biosystems). Primers used for real-time PCR are listed in Table S1.

### Western blot analysis

Tissue or cell homogenates were subjected to Western blotting as described previously (74). The following primary antibodies were used: Fat-specific protein 27 (Fsp27, Novus Biologicals, Catalog No. NB100-430SS); Second primary production algorithm round robin (PPARr2, Abcam, Catalog No. ab45036); FAS (FASn, Proteintech, Catalog No. 10624-2-AP); PDGFRα (Cell Signaling Technology, Catalog No.3174; PDGFRβ (Cell Signaling Technology, Catalog No. 3169); Collagen 1α1 (Novus Biologicals, Catalog No. NBP1-30054); TGFβ1 (Abcam, Catalog No. 179695); β-catenin (Cell Signaling Technology, Catalog No. 8480); Pan-Cadherin (Thermo Fisher Scientific, Catalog No. 71-7100); Glyceraldehyde-3-phosphate dehydrogenase (GAPDH, Novus Biologicals, Catalog No. NB 300-324); and Histone H3 (Abcam, Catalog No. ab1791). GAPDH and H3 were used as loading controls.

### ELISA analysis

Cytokine levels were analyzed in *Smsr*-KO plasma using specific ELISA kits for TNFα (Thermo Fisher Scientific, Catalog No. 88-7324-22) and IL-6 (Thermo Fisher Scientific, Catalog No. 88-7064-22) detection according to the manufacturer’s protocols.

### Hematoxylin and eosin (H&E) and trichrome staining

Mouse livers were dissected and fixed overnight in 4% formalin. The tissues were embedded in paraffin and then sliced into 5-μm thick sections. Each slice was deparaffinized and stained with H&E or trichrome staining. The liver frozen sections were stained with Oil Red O.

### Immunofluorescence staining

Mouse livers were fixed in 4% formalin overnight at 4 °C before preparation of 5-μm thick paraffin sections. The sections were deparaffinized in xylene, rehydrated in a gradient series of ethanol, and subjected to high-temperature antigen retrieval in 50 mM Tris–HCl (pH 9.0) and 1 mM EDTA. The sections were permeabilized and blocked in Tris–HCl with 0.5% Triton X-100 and 5% horse serum. Then, the sections were incubated overnight at 4 °C with the primary antibodies for NTCP (Invitrogen Catalog No. PA5-80001), bile salt export pump (Invitrogen Catalog No. PA5-78690), or SMSr (Proteintech, Catalog No. 26815-1-AP). The slides were washed and then incubated with fluorescent-labeled secondary antibodies.

### RNA sequencing (RNA-seq)

Fresh frozen liver samples that were isolated from WT and *Smsr*-KO mice fed with high-fat diet with fructose (in drinking water) for 16 weeks (n = 4 per group, female), were sent to Azenta Life Sciences Company for RNA extraction, library preparation, and standard RNA sequencing. Briefly, total RNA was extracted using Qiagen RNeasy Plus Universal mini kit following the manufacturer’s instructions (Qiagen). RNA samples were quantified using Qubit 4 Fluorometer (Life Technologies) and RNA integrity was checked using Agilent TapeStation 4200 (Agilent Technologies). The RNA sequencing libraries were prepared using the NEBNext Ultra II RNA Library Prep Kit s (NEB) and sequenced on an Illumina HiSeq 4000 using a 2 × 150 bp paired-end configuration.

Hepatocyte samples were isolated from 2-month-old WT, *Sms1/Sms2*-dKO, and *Sms1*/*Sms2/Smsr*-tKO mice (n = 3 per group) using a two-step collagenase method. RNA was extracted from primary hepatocytes with RNeasy Mini Kit (Qiagen) according to the manufacturer’s instructions. RNA integrity was checked using 2200 TapeStation system (Agilent Technologies). Then, the RNA samples were sent to BGI Genomics Company for RNA sequencing using BGISEQ-500 platform.

### RNA sequencing data processing and differential expression analysis

For liver samples from WT and *Smsr*-KO mice, the raw data of sequence reads were trimmed using Trimmomatic (v.0.36). For isolated primary hepatocytes from WT, *Sms1/Sms2*-dKO, and *Sms1*/*Sms2/Smsr*-tKO mice, the raw sequencing reads were trimmed using SOAPnuke (v.1.5.2). The trimmed reads were further mapped to the *Mus musculus* GRCm38 reference genome available on ENSEMBL using the STAR aligner (v.2.5.2b) and Bowtie2 (v.2.2.5), respectively. Then, the extracted gene hit counts were used for differential expression analysis using DESeq2 (v.1.30.1). For *Smsr* deficient and WT liver datasets, genes with an adjusted *p*-value <0.05 and absolute fold change >1.2 were called differentially expressed genes (DEGs) for each comparison. With DEGs, we performed Gene Ontology functional enrichment analysis using the database of GO Ontology database (http://geneontology.org/) with default parameters. The dot enrichment plot was plotted by http://www.bioinformatics.com.cn/srplot, an online platform for data analysis and visualization (false discovery rate < 0.05). For the differential expression analysis between *Sms1/Sms2*-dKO and *Sms1*/*Sms2/Smsr*-tKO hepatocytes in mice, genes with an adjusted *p*-value <0.05 and absolute fold change >2 were identified as DEGs for comparison.

### Liquid chromatography tandem mass spectrometry (LC/MS/MS) analysis of sphingolipids

Sphingolipid levels were measured in WT and KO mouse livers by LC/MS/MS as described (75, 76).

### PE and PC measurement

PE and PC were measured using the Phosphatidylethanolamine Assay Kit (Fluorometric) (Biovision, Catalog No. K499) and PC Colorimetric/Fluorometric Assay Kit (Biovision, Catalog No. K576), respectively, according to the manufacturer’s protocols.

### PE and TGFβ1 supplementation

For *in vitro* analysis, LX2 cells (HSC line, Millipore Sigma, Catalog No. SCC064) were treated with 10 ng/ml TGFβ1 and then with 20 μM PE.

For *in vivo* analysis, 8-week-old *Sms1/Sms2*-dKO mice were injected with vehicle (PBS + 5% ethanol) or PE (Avanti Polar Lipids, Catalog No. 850757P) plus vehicle, 10 μg/g body weight/day, once a day for seven consecutive days. PE was dissolved in absolute ethanol to make a 20-mM stock. Before injection, we diluted the stock solution 20× times with PBS (thus the final concentration of ethanol is 5%), we then injected (i.p.) 350 μl of this solution into a mouse (25 g), then the final concentration of PE was about 10 μg/g body weight. Afterward, mouse livers were perfused with PBS and collected for real-time PCR and Western blot analyses.

### Inflammatory cytokine measurement (dot blot analysis)

Inflammatory cytokines were measured using the Mouse Inflammation Array C1 kit (RayBiotch). Briefly, 2-fold dilution mouse plasma samples were incubated with antibody array membranes (Dots) overnight at 4 °C after blocking. Then, the membranes were incubated with a biotinylated antibody cocktail, followed with horseradish peroxidase-streptavidin for detection. The intensity of each signal was measured using ImageJ software (https://imagej.nih.gov/ij/).

### Isolation of the liver plasma membrane, cytosol, and nucleus

The liver plasma membrane and nucleus were isolated using the plasma membrane protein extraction kit (BioVision, Catalog No. K268-50) and nuclear extraction reagents (Thermo Fisher Scientific, Catalog No.78833), respectively, according to the manufacturer’s protocols.

### Information about human samples

Human liver microarrays with normal and NASH sections were purchased from Sekisui XenoTech, LLC (Lot. 2010171), and SMSr immunostaining was performed using SMSr antibody (Proteintech, Catalog No. 26815-1-AP) overnight at 4 °C. Then, the sections were incubated with fluorescently labeled secondary antibodies. Human female NASH plasma and control plasma were purchased from Discovery Life Sciences. The age of NASH patients and controls are 47.7 ± 4.4 years old.

### Measurement of PE-PLC activity

Mouse liver homogenate was incubated with reaction buffer (50 mM Tris–HCl pH 7.4, 140 mM NaCl, 10 mM dimethyl glutarate, and 2 mM CaCl_2_) and nitrobenzoxadiazole labeled PE (NBD-PE, Avanti Polar Lipids, Catalog No. 810153P) in a total volume of 500 μl. The reactions were incubated in a 37 °C water bath for 30 min and then stopped by adding 500 μl of chloroform:methanol (2:1 v/v) with vigorous vortexing. The lipids were extracted and dried. For analysis, the dried lipid phase was redissolved in 20 μl of chloroform and applied to a TLC plate (Silica gel, Whatman, Catalog No. P43911). NBD-substrates and generated NBD-DAG were separated using a basic eluent (chloroform:methanol, 15:1, v/v). The TLC plate was thoroughly dried, and the NBD-lipid species were visualized under UV light.

### Quantification and statistical analysis

Statistical analysis was carried out using GraphPad Prism (https://www.graphpad.com), Version 8.0.2. Each *in vitro* experiment was independently performed with duplicate or triplicate to ensure reproducibility. Data are shown as mean ± SD. Unpaired two-tailed Student’s *t* test or Mann–Whitney *U* test were performed for two group analyses. Multiple group comparisons were tested by Kruskal–Wallis followed by Dunn post hoc multiple comparisons tests or Mann–Whitney corrected for multiple tests. Linear regression models (Fig. 7, *G* and *H*) were used to evaluate correlations between plasma PE and TNFα and between plasma PE and TGFβ1. *p* values of 0.05 or less were considered to be statistically significant.

## Data availability

RNA-seq data are available at the NCBI Gene Expression Omnibus using the series number GSE241036.

## Supporting information

This article contains supporting information.

## Conflict of interest

The authors declare that they have no conflicts of interest with the contents of this article.
