# Supplementary material for: Sphingomyelin synthase–related protein SMSr is a phosphatidylethanolamine phospholipase C that promotes nonalcoholic fatty liver disease
Source: J Biol Chem. 2023 Aug 14;299(9):105162. doi: 10.1016/j.jbc.2023.105162 (PMC10494463; doi:10.1016/j.jbc.2023.105162)
Supplement: Supporting Figures S1–S9 and Table S1 [file mmc1.pdf]

Supplementary Materials

Title: Sphingomyelin synthase–related protein, as a phosphatidylethanolamine phospholipase C, deficiency attenuates nonalcoholic fatty liver diseases

Authors: Yeun-po Chiang, Zhiqiang Li, Mulin He, Quiana Jones, Meixia Pan, Xianlin Han, and Xian-Cheng Jiang

A list of contents

1) Table 1
2) Figures

Table S1. Primers for real-time PCR used in this study

Mouse PDGFRα	Forward: 5'-TATCCTCCCAAACGAGAATGAGA-3'<br>Reverse: 5'-GTGGTTGTAGTAGCAAGTGTACC-3'
Mouse PDGFRβ	Forward: 5'-TTCCAGGAGTGATACCAGCTT-3'<br>Reverse: 5'-AGGGGGCGTGATGACTAGG-3'
Mouse Col1α1	Forward: 5'-GCTCCTCTTAGGGGCCACT-3'<br>Reverse: 5'-CCACGTCTCACCATTGGGG-3'
Mouse TIMP1	Forward: 5'-GCAACTCGGACCTGGTCATAA-3'<br>Reverse: 5'-CGGCCCGTGATGAGAAACT-3'
Mouse Ccnd2	Forward: 5'-GAGTGGGAACTGGTAGTGTTG-3'<br>Reverse: 5'-CGCACAGAGCGATGAAGGT-3'
Mouse Myct1	Forward: 5'-ATGGCTAATAACACCACGAGC-3'<br>Reverse: 5'-CAGCGCCCAGAGAAATCCT-3'
Mouse TGFβ1	Forward: 5'-CTCCCGTGGCTTCTAGTGC-3'<br>Reverse: 5'-GCCTTAGTTTGGACAGGATCTG-3'
Mouse FSP27	Forward: 5'-CAGAAGCCAACTAAGAAGATCG-3'

	Reverse: 5’-TGTAGCAGTGCAGGTCATAG-3’
Mouse CD36	Forward: 5’-ATTGGTCAAGCCAGCT-3’<br>Reverse: 5’-TGTAGGCTCATCCACTAC-3’
Mouse GAPDH	Forward: 5’-AGGTCGGTGTGAACGGATTTG-3’<br>Reverse: 5’-TGTAGACCATGTAGTTGAGGTCA-3’
Human PDGFRα	Forward: 5’-TGGCAGTACCCCATGTCTGAA-3’<br>Reverse: 5’-CCAAGACCGTCACAAAAAGGC-3’
Human PDGFRβ	Forward: 5’-AGCACCTTCGTTCTGACCTG-3’<br>Reverse: 5’-TATTCTCCCGTGTCTAGCCCA-3’
Human Col1α1	Forward: 5’-GAGGGCCAAGACGAAGACATC-3’<br>Reverse: 5’-CAGATCACGTCATCGCACAAC-3’
Human TIMP1	Forward: 5’-CTTCTGCAATTCCGACCTCGT-3’<br>Reverse: 5’-ACGCTGGTATAAGGTGGTCTG-3’
Human Ccnd2	Forward: 5’-ACCTTCCGCAGTGCTCCTA-3’<br>Reverse: 5’-CCCAGCCAAGAAACGGTCC-3’
Human Myct1	Forward: 5’-CAATCGGGCTGGTACTTGGAG-3’<br>Reverse: 5’-CGTGGGTGTAAGAAGACCTAGA-3’
Human Mycn	Forward: 5’-ACCCGGACGAAGATGACTTCT-3’<br>Reverse: 5’-CAGCTCGTTCTCAAGCAGCAT-3’
Human TGFβ1	Forward: 5’-GGCCAGATCCTGTCCAAGC-3’<br>Reverse: 5’-GTGGGTTTCCACCATTAGCAC-3’
Human SMS1	Forward: 5’-TGTGCCGAGTCTCCTCTGA-3’<br>Reverse: 5’-CCGTTCTTGTGTGCTTCCAAA-3’
Human SMS2	Forward: 5’-CAAATTGCTATGCCCACTGAATC-3’<br>Reverse: 5’-GTTGTCAAGACGAGGTTGAAAAC-3’

Figures

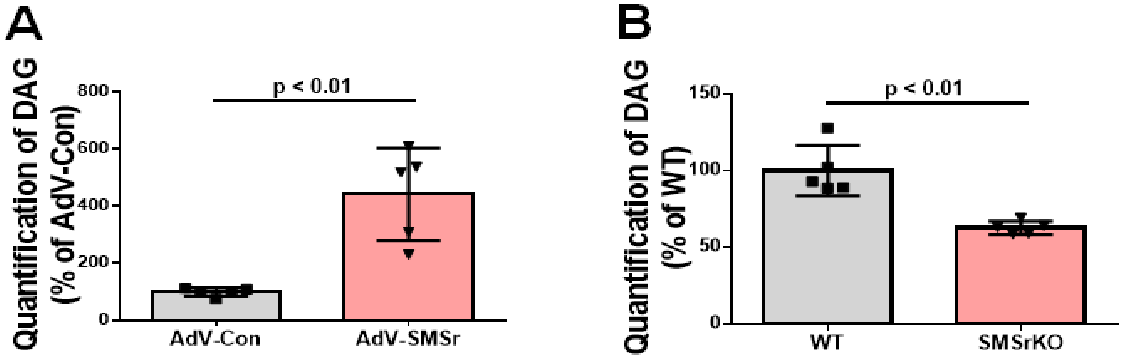

Figure S1. PE-PLC activity measured by the formation of NBD-diacylglyceride (DAG). (A） AdV-mediated SMSSr/PE-PLC expression; B） SMSr deficiency. Data are presented as mean ± SD, n =5 per group. The p values were calculated by Mann-Whitney *U* test.

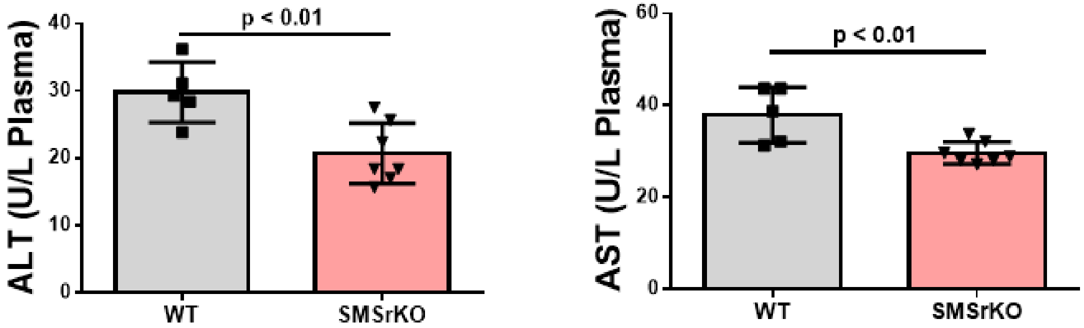

Figure S2. Alanine transaminase (ALT) and aspartate transaminase (AST) measurements in WT and SMSr-deficient mouse plasma. Data are presented as mean ± SD, n =5-7 per group. The p values were calculated by Mann-Whitney *U* test.

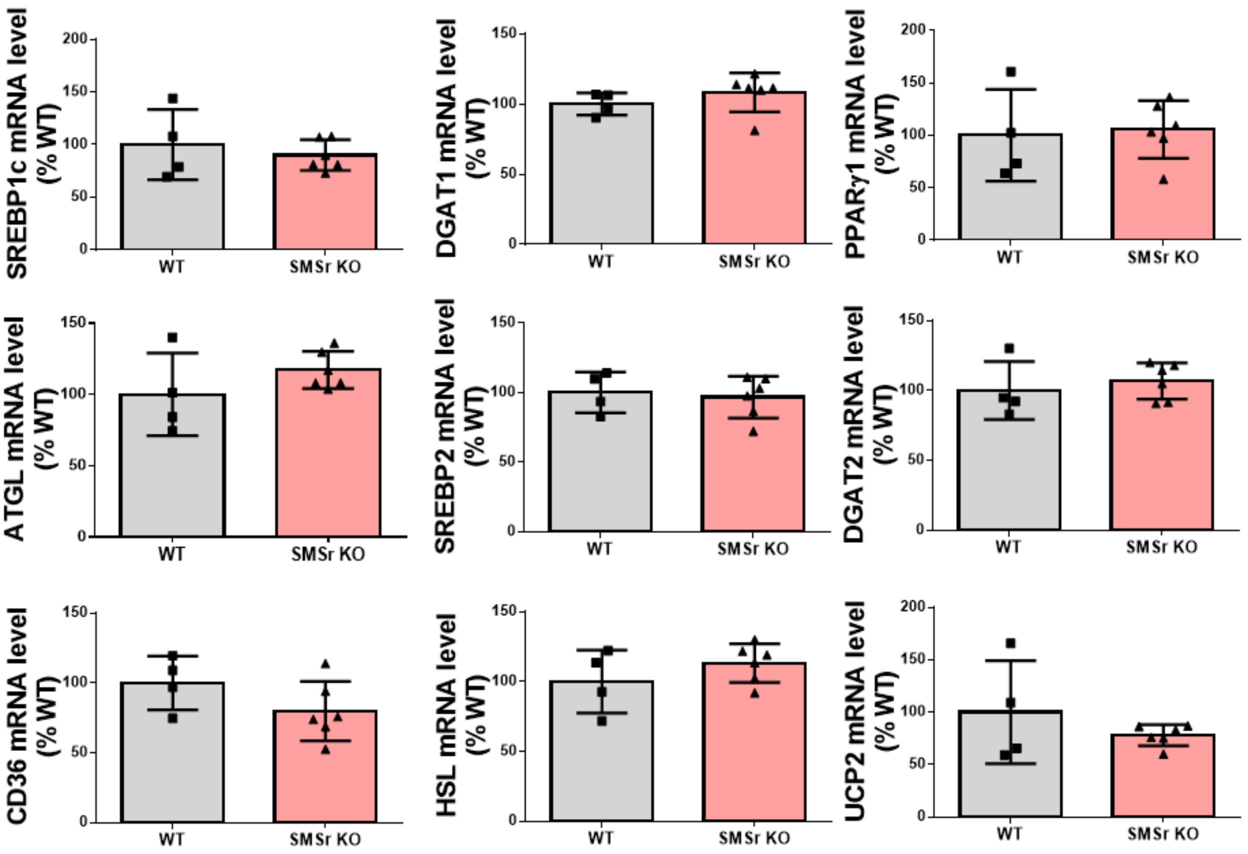

Figure S3. Real-time PCR analysis (WT vs SMSr-deficient mice). Data are presented as mean ± SD, n =4-6 per group. The p values were calculated by Mann-Whitney *U* test.

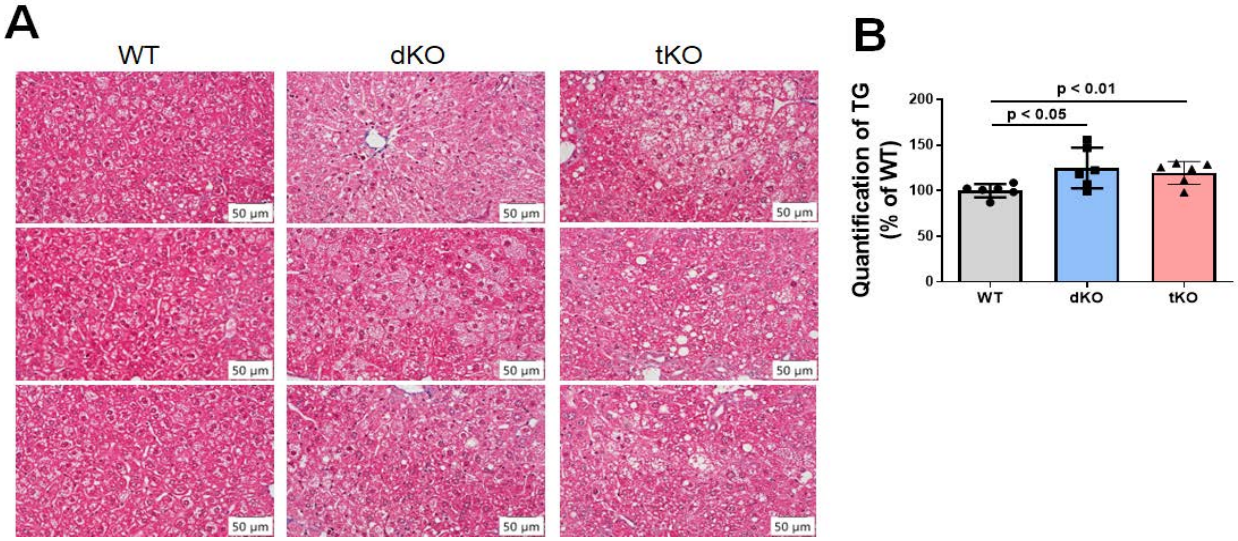

Figure S4. Trichrome blue staining mouse liver sections. WT and SMSr-deficient mice (three month old) were fed with a high-fat diet for 6 weeks. Their liver sections were stained with trichrome blue (A) and their liver triglyceride was measured (B). Data represent 6 mice/group.

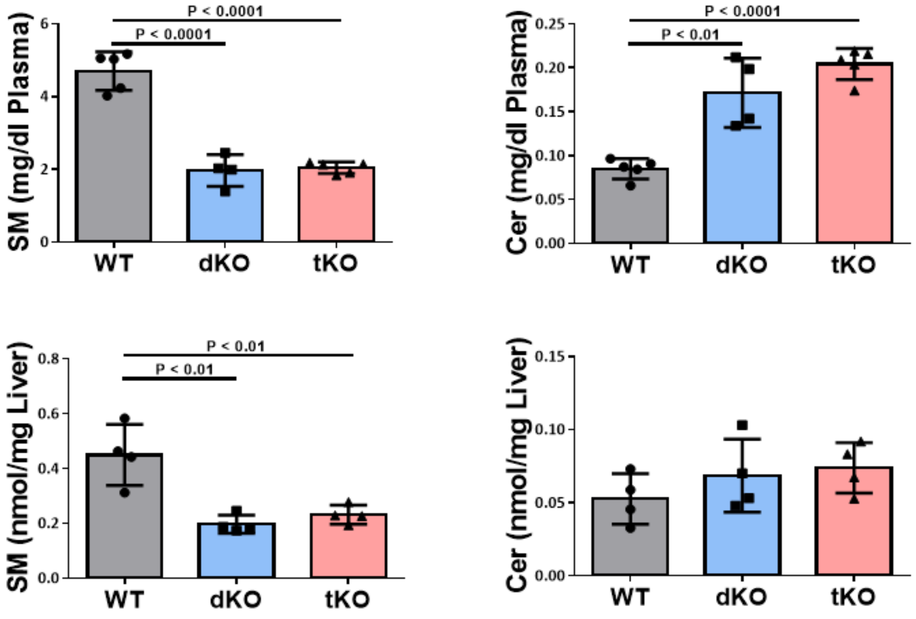

Figure S5. Measurement of SM and ceramide in WT and SMSr-deficient mouse plasma and liver. WT and SMSr-deficient mice were on chow diet. Data represent mean ± SD, *n*=4–5. Data are presented as mean ± SD, n = 4-5 per group. The p values were calculated by Kruskal-Wallis test followed by Mann-Whitney pairwise tests.

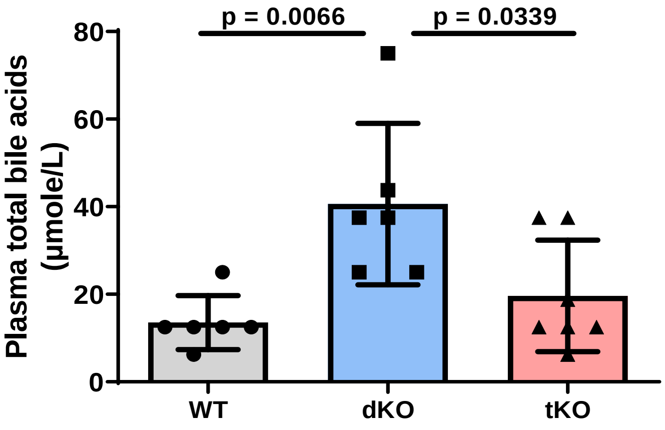

Figure S6. Plasma total bile acid measurement. Six-month-old mice were on chow diet were used. Data represent mean ± SD, *n*=6–7. The p values were calculated by Kruskal-Wallis test followed by Mann-Whitney pairwise tests.

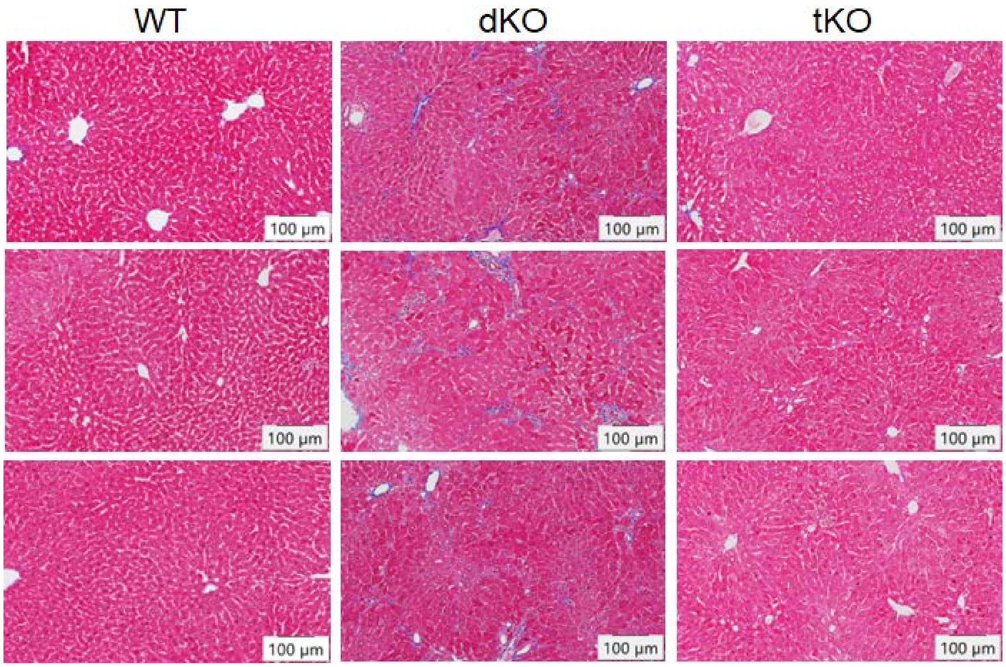

Figure S7. Liver section trichrome blue staining. Mice were on chow diet. Liver sections were prepared from WT, *Sms1*/*Sms2*-dKO and *Sms1*/*Sms2/Smsr*-tKO mice and stained with trichrome blue. Data represent 5 mice/group.

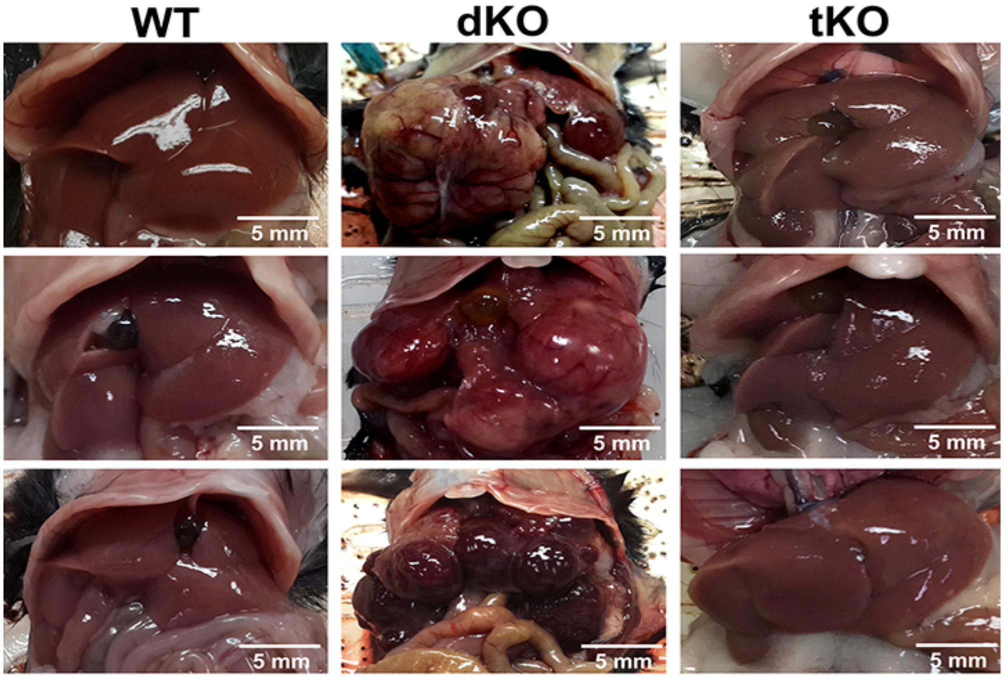

Figure S8 Tumor formation. One year old WT, *Sms1*/*Sms2*-dKO and *Sms1*/*Sms2/Smsr*-tKO mice, on chow diet, were sacrificed and their abdominals were opened and livers were photographed. Data represent 6 mice/group. All the dKO mice(6/6) developed tumor with various sizes and none WT and the tKO mice had tumor.

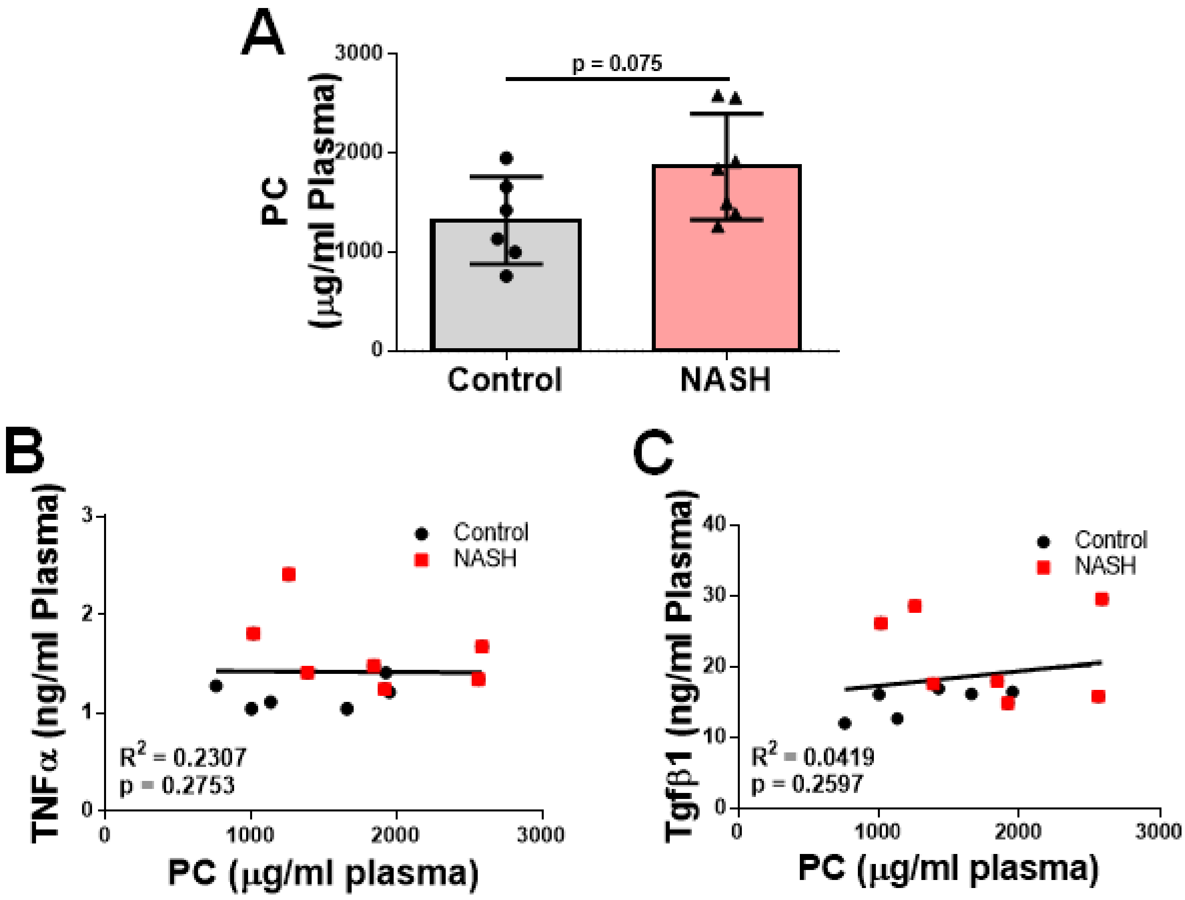

Figure S9. Phosphatidylcholine (PC) levels in NASH patients. (A) Plasma PC levels were measured and p value was calculated by unpaired two-tailed Student's *t* test, n-6-7. (B) Correlation between human plasma PC and TNFa levels. (C) Correlation between human plasma PC and TGFb1 levels. The p values of were calculated by linear regression model.
